# *Ramulus Mori* (Sangzhi) Alkaloids Alleviate Diabetic Nephropathy through Improving Gut Microbiota Disorder

**DOI:** 10.3390/nu16142346

**Published:** 2024-07-20

**Authors:** Wenxiu Liu, Saijun Xu, Bin Zhang, Xiaobo Sun

**Affiliations:** 1 Institute of Medicinal Plant Development, Peking Union Medical College, Chinese Academy of Medical Sciences, Beijing 100193, China; liuwenxiu1sdu@163.com (W.L.); xsj365fighting@163.com (S.X.); 2 Key Laboratory of Bioactive Substances and Resources Utilization of Chinese Herbal Medicine, Ministry of Education, Beijing 100193, China; 3 Diabetes Research Center, Chinese Academy of Medical Sciences, Beijing 100193, China; 4 Key Laboratory of Efficacy Evaluation of Chinese Medicine against Glyeolipid Metabolism Disorder Disease, State Administration of Traditional Chinese Medicine, Beijing 100193, China

**Keywords:** diabetic nephropathy, Sangzhi alkaloids, gut microbiota, metabolite, pentadecanoic acid

## Abstract

Diabetic nephropathy (DN), one of the leading causes of end-stage kidney failure worldwide, is closely associated with high mortality in diabetic patients. However, therapeutic drugs for DN are still lacking. *Ramulus Mori* alkaloids (SZ-A), an effective component of alkaloids extracted from *Ramulus Mori*, have been found to improve glucose and lipid metabolism to mitigate diabetes and obesity; however, few studies have focused on their effects on DN progression. Thus, we investigated the protective role of SZ-A on DN through 16S rRNA sequencing, non-targeted metabolomics, and fecal microbiota transplantation (FMT) experiments. To address our hypothesis, we established the DN mouse model by combining a high-fat diet (HFD) with streptozotocin (STZ) injection. Herein, we demonstrated that SZ-A supplementation was recalcitrant to renal injury in DN mice, improving glomerular morphology, reversing the blood biochemistry parameters, and ameliorating podocyte injury. Importantly, the composition of the gut microbiota altered after SZ-A treatment, especially with the elevated abundance of *Dubosiella* and the increased level of serum pentadecanoic acid. FMT experiments further revealed that the gut microbiota exerted critical effects in mediating the beneficial roles of SZ-A. In vitro experiments proved that pentadecanoic acid administration improved podocyte apoptosis induced by AGEs. Taken together, SZ-A play a renoprotective role, possibly through regulating the gut microbiota and promoting pentadecanoic acid production. Our current study lends support to more extensive clinical applications of SZ-A.

## 1. Introduction

Diabetic nephropathy (DN) is a common complication of diabetes mellitus that occurs in approximately one-third of patients with type 1 and type 2 diabetes [1]. In clinical settings, DN is characterized by a high level of albuminuria and a low rate of glomerular filtration and is tightly related to microvascular kidney damage. Currently, the intervention strategies for DN mainly concentrate on managing hyperglycemia, hypertension, hyperlipidemia, and obesity [2,3]. Nevertheless, these approaches only address the control of blood glucose and blood pressure to alleviate its complications, not the DN itself. Thus, finding drugs that can effectively cure diabetes-induced kidney damage is critical for DN treatment.

Clinical trials have provided credible evidence for the use of traditional Chinese medicines against DN. The branch of *Morus alba* L., known as *Ramulus Mori* (Sangzhi), enriches abundant chemical components [4,5]. Among them, *Ramulus Mori* alkaloids (SZ-A) have been approved by the Food and Drug Administration of China for the treatment of type 2 diabetes mellitus [6,7]. Current preclinical studies about SZ-A are still ongoing, but most focus on the pharmacological activities of hypoglycemic and hypolipidemic drugs. A study on type 2 diabetes discovered that SZ-A mitigate the overall metabolic profile, especially glucose and lipid metabolism, in diabetic KKAy mice [8]. Another study suggested that SZ-A attenuate inflammation caused by lipid metabolism disorders [9]. DN, as a complication of diabetes, shares a similar mechanism with it, implying the potential effects of SZ-A on DN. However, the beneficial effect of SZ-A on DN is still undefined, and the mechanism is also still unclear.

Compelling evidence has demonstrated that gut microbiota is associated with type 2 diabetes and DN, and fecal microbiota transplantation (FMT) is an effective way to alleviate metabolic disease [10,11]. It has been reported that dietary fiber supplements could prevent patients from DN development via boosting short-chain fatty acid (SCFA)-producing bacteria [12,13]. Furthermore, DN is also influenced by the metabolites that the host and gut bacteria produce. Hu et al. found depletion of the gut microbiota is accompanied by a reduction of serum acetate levels, which is positively associated with cholesterol levels in the kidney. A further investigation revealed that the FMT experiment could successfully lower serum acetate levels and lessen tubulointerstitial injury in diabetic rats by suppressing the disturbance of cholesterol homeostasis [14,15]. These findings indicated that the gut microbiota and its related metabolism are critical mediators involved in the progression of DN. Interestingly, it has been reported that SZ-A regulate the composition of the gut microbiota in obese mice and KKAy mice [8,16]. Investigation into the relationship among SZ-A, gut microbiota, and DN is deserved.

The present work aimed to confirm the protective effect of SZ-A on DN as well as investigate the underlying mechanism based on 16S rRNA gene sequencing and non-targeted metabolomics. To some extent, this study not only provides complementary therapy for DN treatment but also points out further directions for the mechanism exploration of DN drugs.

## 2. Materials and Methods

### 2.1. Animal Studies

Eight-week-old C57BL/6J male mice were purchased from SPF (Beijing) Biotechnology Co., Ltd. (Beijing, China). They were fed at a constant temperature (22 ± 2 °C), humidity (56 ± 5%), a 12 h light–dark cycle, and adequate food and water. All the animal experiments were approved by the Ethical Committee on Animal Experimentation of the Chinese Academy of Medical Sciences and Peking Union Medical College (ethical code: SLXD-20231031021). After one-week adaptive feeding, a high-fat diet (HFD) or normal diet was given for 4 weeks. Then the mice fed with HFD were intraperitoneally injected with a dose of 140 mg/kg streptozotocin (STZ) (Sigma-Aldrich, Saint Louis, MO, USA) to establish the DN model. After the model was successfully established, all the mice were randomly divided into a control group, model group, positive control group (Acarbose, Bayer AG, Leverkusen, Germany, 50 mg/kg, i.g.), and SZ-A treatment group (SZ-A, Beijing Wehand-Bio Pharmaceutical Co., Ltd., Beijing, China, 50 mg/kg and 100 mg/kg, i.g.). The administration lasted for 8 weeks. The mice in the control group were fed with a normal diet, and the other mice were unceasingly fed with HFD.

For the mice used in the fecal microbiota transplantation (FMT) experiment, when the DN model established by HFD/STZ was successful, the mice were divided into C + FMT-PBS, M + FMT-PBS, M + FMT-M, and M + FMT-SZ-A groups, respectively. After one week of antibiotic treatment, the fresh feces from the model group and the SZ-A high-dose group were collected in a sterile environment every day for the day’s FMT experiment. The FMT experiment lasted for 3 weeks.

### 2.2. Cell Culture

The cell line of mice podocyte MPC5 cells (BNCC342021) was obtained from BNCC and cultured in DMEM (GIBCO BRL, Grand Island, NY, USA) containing 10% FBS (GIBCO BRL, Grand Island, NY, USA) and 1% ampicillin and streptomycin (GIBCO BRL, Grand Island, NY, USA) at a temperature of 37 °C in a humidified atmosphere with 5% CO_2_.

To establish the podocyte injury model of DN in vitro, MPC5 cells were cultured in 96-well plates or 12-well plates for 24 h and then cultured in DMEM containing 200 μg/mL AGEs (Bioss, Woburn, MA, USA) for 24 h. Pentadecanoic acid (Solarbio, Beijing, China) was added to the medium at the same time. Cell Counting Kit-8 (GLPBIO, Montclair, CA, USA) and TUNEL Staining Kit (Beyotime, Shanghai, China) were used to detect cell viability and apoptosis, respectively.

### 2.3. Blood and Urine Biochemical Parameters

The level of fasting blood glucose (FBG) was measured by a blood glucose meter (Roche, Basel, Switzerland) after fasting. The levels of serum triglyceride (TG), total cholesterol (TCH), uric acid (UA), serum creatinine (SCr), blood urea nitrogen (BUN), beta-2-microglobulin (β2-MG), high-density lipoprotein (HDL), low-density lipoprotein (LDL), kidney injury molecule-1 (KIM-1), and urinary albumin (UAlb) were measured using the Beckman Coulter AU480 automatic biochemical analyzer as described in the protocols of the testing kits (Biosano, Beijing, China).

### 2.4. Histopathology

After the mice sacrifice, we immediately fixed the left kidney tissues with 4% paraformaldehyde, and then the fixed kidney tissues were embedded in paraffin to make sections for hematoxylin and eosin (H&E) staining and PAS staining. Subsequently, we assessed the renal section changes with the microscope (LeicaFS1000, Wetzlar, Germany).

### 2.5. 16S rRNA Sequencing Analysis

Samples of mouse feces were collected and frozen in a refrigerator at −80 °C. Fecal genomic DNA was extracted and detected with 1% agarose gel electrophoresis. We processed each sample with a PCR instrument (GeneAmp 9700, ABI, Foster, CA, USA), and the products were extracted from the gel by using the AxyPrep DNA Gel Recovery Kit (Axygen, Tewksbury, MA, USA). They were then analyzed and mixed in appropriate proportions according to sequencing requirements. Illumina’s Miseq platform for PE300 was utilized. Sequencing reads were demultiplexed, quality controlled by fastp (version 0.21.0), and merged by FLASH (version 1.2.7), and then imported into QIIME2 (version 2022.8). We used the DADA2 algorithm to filter and denoise sequences while removing the chimeric sequences. After that, the features table was generated to obtain the representative sequence of ASV. Next, through the comparison of species annotations in the database, the characteristic statistical tables of boundary, phylum, class, order, family, genus, and species classification annotation information were gained. And finally, we conducted analyses of community composition, alpha diversity, beta diversity, and function prediction.

### 2.6. Untargeted Metabolomics Analysis

After taking blood from the eyeballs of mice, centrifuge at 3000 rpm for 15 min, and take the supernatant as serum. One hundred microliters of serum samples were resuspended with pre-cooled 80% methanol by vortexing. After incubation on ice for 5 min, 15,000× *g* was centrifuged at 4 °C for 20 min. After dilution, 15,000× *g* was centrifuged at 4 °C for 20 min, and the supernatant was injected into the liquid chromatography–tandem mass spectrometry (LCMS/MS) system for analysis [17,18], which was performed by a Thermo Syncronis C18 (2.1 mm × 100 mm, 1.7 µm) UHPLC system (ThermoFisher, Regensburg, Germany) and an Orbitrap Q ExactiveTM series mass spectrometer (Thermo Fisher, Germany). Approximately 0.1% FA in water and acetonitrile were used as eluents A and B, respectively. After that, MS analysis was carried out in the positive/negative polarity mode or the positive/negative auto-switching mode.

The raw data generated by UHPLC-MS/MS were analyzed using TraceFinder 3.2.0 (ThermoFisher). To acquire accurate qualitative and relative quantitative results, we matched the peaks of every metabolite with the mzCloud (https://www.mzcloud.org/, accessed on 26 July 2021) and self-built databases. Statistical analysis was carried out using statistical software R (R version R-3.4.3), Python (Python version 2.7.6), and CentOS (CentOS release 6.6). 

### 2.7. FMT Experiment

The FMT experiment was performed according to the published studies [19,20]. Ampicillin (Macklin, Shanghai, China), neomycin sulfate (Macklin, Shanghai, China), metronidazole (Macklin, Shanghai, China), and vancomycin (Macklin, Shanghai, China) were dissolved in sterile water, and the concentrations of intragastric administration were 40 mg/kg, 40 mg/kg, 40 mg/kg, and 20 mg/kg, respectively. These antibiotics were given for 7 days to the recipient mice. The fecal fluid for the FMT test was freshly prepared every day. One hundred milligrams of feces collected from donor mice was suspended in 1 mL of sterile PBS solution and then centrifuged at 800× *g* for 3 min. Each recipient mouse was given 200 μL of supernatant by gavage. Among them, normal mice and DN mice were treated with antibiotics and then given PBS solution by gavage, which were the C + FMT-PBS group and the M + FMT-PBS group, respectively. DN mice receiving fecal fluid from DN mice were in the M + FMT-M group, and DN mice receiving fecal fluid from DN mice treated with a high dose of SZ-A were in the M + FMT-SZ-A group.

### 2.8. Transmission Electron Microscope (TEM)

After sacrifice, the right kidney samples were fixed in the electron microscope fluid, washed with 0.2 M phosphate buffer, fixed with 1% osmium tetroxide, washed again, dehydrated with an alcohol gradient (30%, 50%, 60%, 70%, 80%, 90%, 95%, 100%), replaced with alcohol acetone, and soaked with an embedding agent gradient (1:2; 1:1). After embedding with pure resin (812#), ultra-thin sections were prepared (Leica, Wetzlar, Germany) and stained with uranyl acetate and lead citrate. Finally, we observed the ultrastructure of kidney tissues by TEM (Hitachi, Tokyo, Japan).

### 2.9. Statistical Analysis

Statistical analysis was performed by the software GraphPad Prism 8.0. Experimental data were subjected to a homogeneity test of variances, and Student’s unpaired t tests were used between the two groups. Comparisons of multiple groups were performed with one-way ANOVA followed by post hoc Tukey’s test. All results were shown as means ± SD, and those with a value of *p* < 0.05 were considered to be statistically significant.

## 3. Results

### 3.1. SZ-A Ameliorate Lipid and Glucose Metabolic Disorders in HFD/STZ-Induced Diabetic Mice

**Figure 1:**
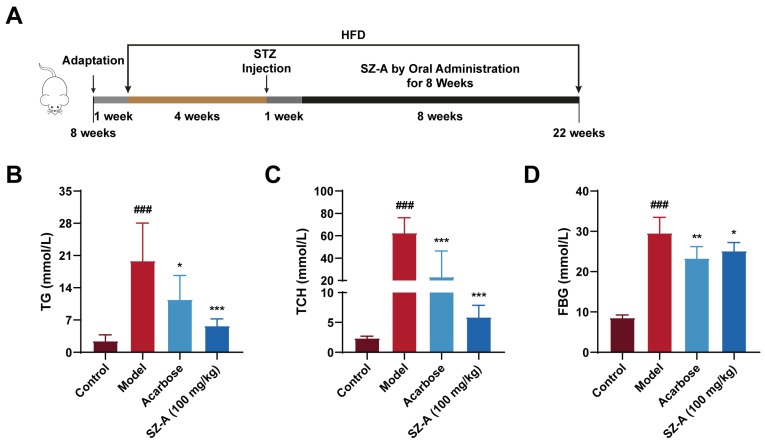
The hypolipidemic and hypoglycemic effects of SZ-A in mice with DN induced by HFD and STZ. (**A**) Procedure for establishment of DN model and administration of SZA. (**B**) Serum TG levels. (**C**) Serum TCH levels. (**D**) Blood glucose levels. The data represent the mean ± SD. n = 6, ### *p* < 0.001 versus the control group; * *p* < 0.05, ** *p* < 0.01, *** *p* < 0.001 versus the model group.

To investigate the positive effects of SZ-A on lipid and glucose metabolic disorders, we fed the animals a high-fat diet and then intraperitoneally injected them with STZ at 4 weeks. After eight weeks of treatment, we detected the relevant indices of lipid and glucose metabolism (Figure 1A). Serum levels of TG and TCH were notably increased in the model group compared to the control group (*p* < 0.0001), whereas administration of 100 mg/kg SZ-A significantly reduced both TG (*p* < 0.0001) and TCH (*p* < 0.0001) (Figure 1B,C). Meanwhile, SZ-A could significantly reduce the increased blood glucose in diabetic mice induced by HFD/STZ (Figure 1D).

### 3.2. SZ-A Prevent Kidney Injury in Diabetic Mice

**Figure 2:**
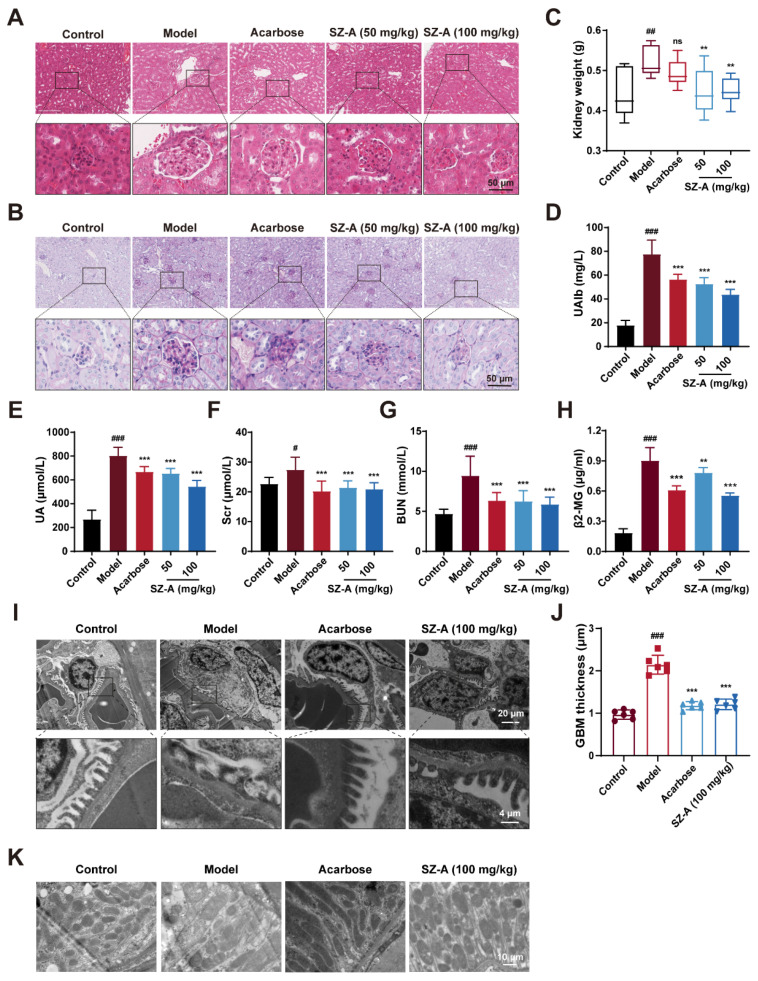
The renoprotection effects of SZ-A in HFD and STZ-induced mice. (**A**) Representative images of hematoxylin–eosin (H&E) staining of kidney tissue sections from each group (scale bar: 50 μm). (**B**) Representative images of PAS staining of kidney tissue sections from each group (scale bar: 50 μm). (**C**) Kidney weight, n = 9–10. (**D**) Urine microalbumin content, n = 9–10. (**E**–**H**) Serum biochemical indexes of mice, n = 9–10. (**I**,**J**) Representative electron microscopy images of mouse glomerulus from each group (scale bars: 20 μm and 4 μm) and TEM quantification of GBM thickness, n = 6. (**K**) Representative electron microscopy images of kidney tissue mitochondria from each group (scale bar: 10 μm). The data represent the mean ± SD. # *p* < 0.05, ## *p* < 0.01, ### *p* < 0.001 versus the control group; ** *p* < 0.01, *** *p* < 0.001 versus the model group.

Next, we examined the protective effect of SZ-A on kidney injury in HFD/STZ-induced diabetic mice. The kidney weight was significantly decreased by SZ-A as compared to the model group (Figure 2C). Urinary microalbumin (UAlb), serum uric acid (UA), SCr, BUN, and beta-2-microglobulin (β2-MG) were representative biochemical indices to reflect renal function. These indices were notably increased in HFD/STZ-induced diabetic mice (*p* < 0.001, *p* < 0.001, *p* < 0.05, *p* < 0.001, and *p* < 0.001, respectively). Treatment with 50 mg/kg or 100 mg/kg SZ-A significantly reversed these changes (*p* < 0.001, *p* < 0.001, *p* < 0.001, *p* < 0.001, *p* < 0.01, respectively), suggesting that SZ-A could ameliorate the kidney injury caused by HFD/STZ (Figure 2C–F). Simultaneously, the kidney sections were subjected to hematoxylin and eosin (H&E) and PAS staining. As Figure 2G,H shows, the kidneys of model mice exhibited abnormal morphology with larger glomeruli and more proliferation of mesangial cells when compared to those of control mice; meanwhile, much more glycogen was found to deposit in the kidney tissues of model mice. SZ-A treatment ameliorated histopathological damage caused by HFD/STZ.

Additionally, we further assessed the changes in the kidney tissue ultrastructure by transmission electron microscopy (TEM). Podocytes are highly specialized cells in the glomeruli and are an important part of the glomerular filtration barrier [21]. During the progression of DN, AGEs and inflammatory factors lead to the destruction of podocyte structural integrity, podocyte foot process fusion, and detachment from the glomerular basement membrane (GBM), causing proteinuria [22,23]. According to the TEM analysis, in model mice, there was foot process fusion and the thickness of GBM increased significantly; however, the foot process fusion was alleviated and GBM thickness was reversed after SZ-A supplement (Figure 2I,J). This result conforms with the data in Figure 2D. In addition, the changes in mitochondria in renal cells of the model group were further observed by TEM. The mitochondria were dissolved and the number was reduced, while the morphology and number of mitochondria were robustly ameliorated after SZ-A treatment (Figure 2K). Recapitulate that SZ-A are verified to have effective therapeutic roles in renal injury, which can counteract the progression of DN induced by HFD/STZ.

### 3.3. SZ-A Alters the Composition of Gut Microbiota in STZ and HFD-Induced Mice

**Figure 3:**
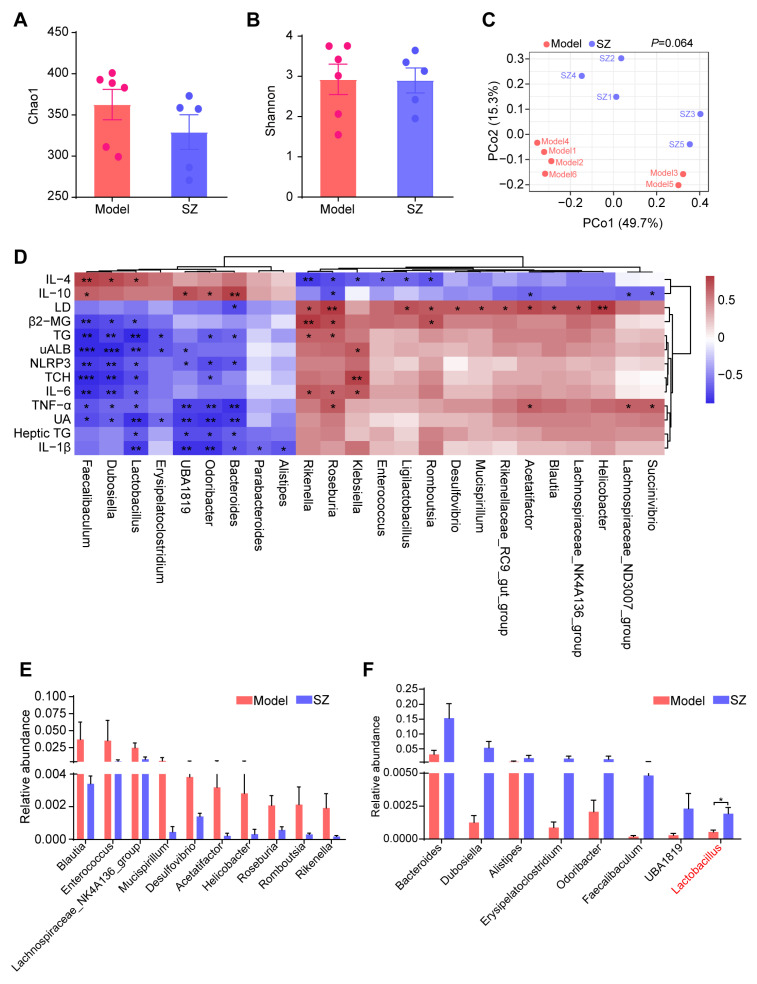
16S rRNA sequencing analysis results of SZ-A-treated DN mice. (**A**) Alpha diversity analysis (the Chao1 index) between groups. (**B**) Alpha diversity analysis (the Shannon index) between groups. (**C**) Principal coordinate analysis (PCoA) diagram of transcriptional profiling of the mouse cecal contents. (**D**) Correlation analysis between gut microbiota and biochemical indicators. (**E**,**F**) Relative abundance of the bacterial altered with SZ-A treatment at the genera level. The data represent the mean ± SD. n = 5–6, * *p* < 0.05, ** *p* < 0.01, *** *p* < 0.001.

Compelling evidence proved that SZ-A could alleviate glucose metabolism via modulating the gut microbiota [8]. We, therefore, monitored the alteration of gut microbiota in STZ/HFD-induced mice to substantiate the potential role of gut microbiota in ameliorating diabetic nephropathy. The alpha diversity of gut microbiota analyzed by Chao1 and Shannon indices showed that there were no obvious differences between the model and SZ group (Figure 3A,B). Similarly, the structure of gut microbiota among the two groups also had no significant separation, which was reflected by the principal coordinate analysis (PCoA) diagram (Figure 3C). We further performed correlation analysis between gut microbiota (relative abundance > 0.001) and biochemical indicators to find genera that are closely related to disease development. As shown in Figure 3D, a total of 24 genera were positively or negatively associated with 13 biochemical indicators. Especially *Faecalibaculum*, *Dubosiella*, *Lactobacillus*, *UBA1819*, *Odoribacter*, and *Bacteroides* were positively related to the anti-inflammatory factors: interleukin 10 (IL-10) and interleukin 4 (IL-4), whereas they are negatively correlated with proinflammatory factors such as interleukin 1β (IL-1β), interleukin-6 (IL-6), tumor necrosis factor alpha (TNF-α), NLRP3, renal function index, such as UA, UAlb, β2-MG, as well as serum TG, serum TCH, and heptic TG (Figure 3D). Simultaneously, some genera like *Rikenella*, *Roseburia*, and *Klebsiella* were positively associated with LD, IL-6, TNF-α, β2-MG, TG, and TCH, while negatively related to IL-10 and IL-4 (Figure 3D). We next identified the abundance change of these genera (relative abundance > 0.001) and found the levels of *Blautia*, *Enterococcus*, *Lachnospiraceae_NL4A136_group*, *Mucispirillum*, *Desulfovibrio*, *Acetatifactor*, *Helicobacter*, *Roseburia*, *Romboutsia*, and *Rikenella* were reduced by SZ-A, but had no statistical difference (Figure 3E). However, the relative abundances of *Bacteroides*, *Dubosiella*, *Alistipes*, *Erysipelatoclostridium*, *Odoribacter*, *Faecalibaculum*, *UBA1819*, and *Lactobacillus* had more than doubled after SZ-A treatment as compared with the model group (Figure 3F). Importantly, *Dubosiella* and *Lactobacillus* had statistical differences between the two groups (*p* < 0.05), suggesting that the alteration of *Dubosiella* and *Lactobacillus* may involve the relief of diabetic nephropathy by SZ-A.

### 3.4. SZ-A Change the Metabolic Profile in Diabetic Mice

**Figure 4:**
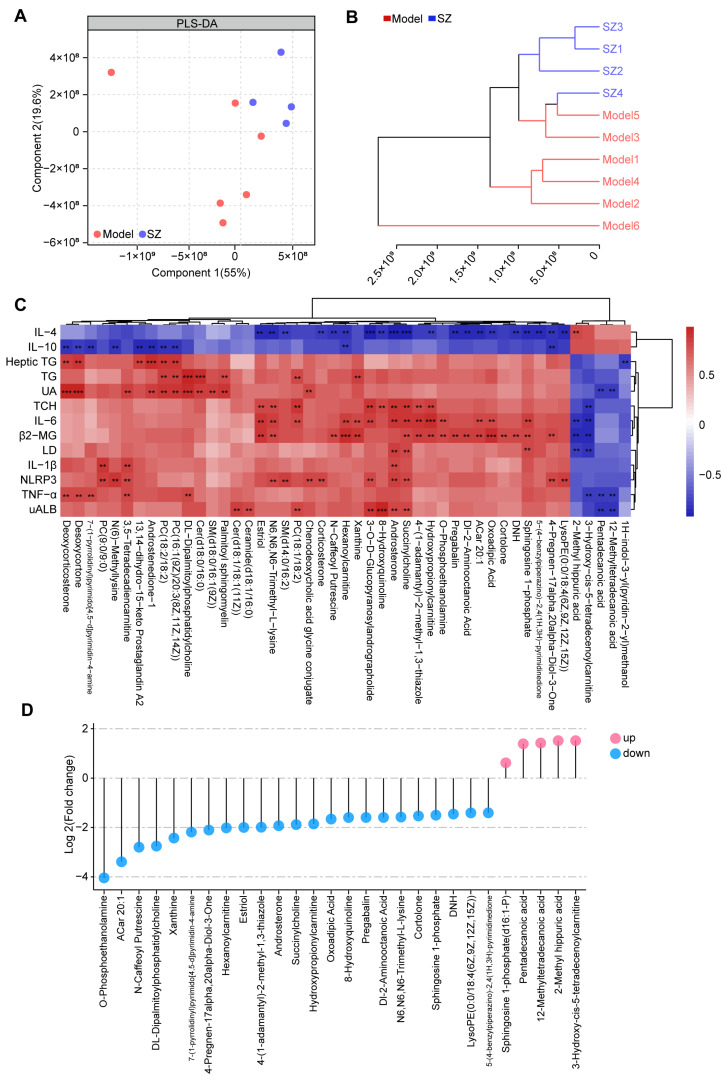
Non-targeted metabolomics analysis results of SZ-A-treated DN mice. (**A**) Partial least-squares discriminant analysis (PLS-DA) of metabolic profiles between groups (model group: n = 6; SZ-A group: n = 4). (**B**) Clustering analysis (bray-curtis distance) of metabolic profiles between groups (model group: n = 6; SZ-A group: n = 4). (**C**) Clustering heat maps and correlation analysis between differential metabolites, red indicates positive correlation and blue indicates negative correlation, ** *p* < 0.01, *** *p* < 0.001. (**D**) The levels of metabolites between groups. Blue plot points represent down-regulated metabolites in the SZ-A group compared to the model group with statistical significance, while red plot points represent up-regulated metabolites with statistical significance.

To further identify the metabolic alteration induced by SZ-A, we analyzed the metabolic profile among two groups by high-performance liquid chromatography-tandem mass spectrometry (HPLC-MS/MS). Both partial least-squares discriminant analysis (PLS-DA) and clustering analysis (bray-curtis distance) showed that the overall metabolic profile in the SZ group was separated from the model group (Figure 4A,B). The correlation analysis based on the Spearman rank correlation coefficient revealed that forty-seven metabolites were negatively or positively related to 13 biochemical indices. Among these, succinylcholine and androsterone were positively associated with UAlb, NLRP3, IL-1β, LD, β2-MG, IL-6, and TCH, whereas they were negatively correlated with IL-4. Additionally, 2-methyl hippuric acid, 3-hydroxy-cis-5-tetradecenoylcarnitine, pentadecanoic acid, and 12-methyltetradecanoic acid (12-MTA) were negatively related to UAlb, TNF-α, LD, β2-MG, IL-6, TCH, and UA, while 2-methyl hippuric acid was positively related to IL-4 (Figure 4C). We then measured the levels of these metabolites among two groups. A total of twenty-three metabolites were notably decreased after SZ-A treatment, but five metabolites were elevated (Figure 4D).

### 3.5. Correlation between Gut Microbiota and Metabolites in SZ-A-Conditioned Mice

**Figure 5:**
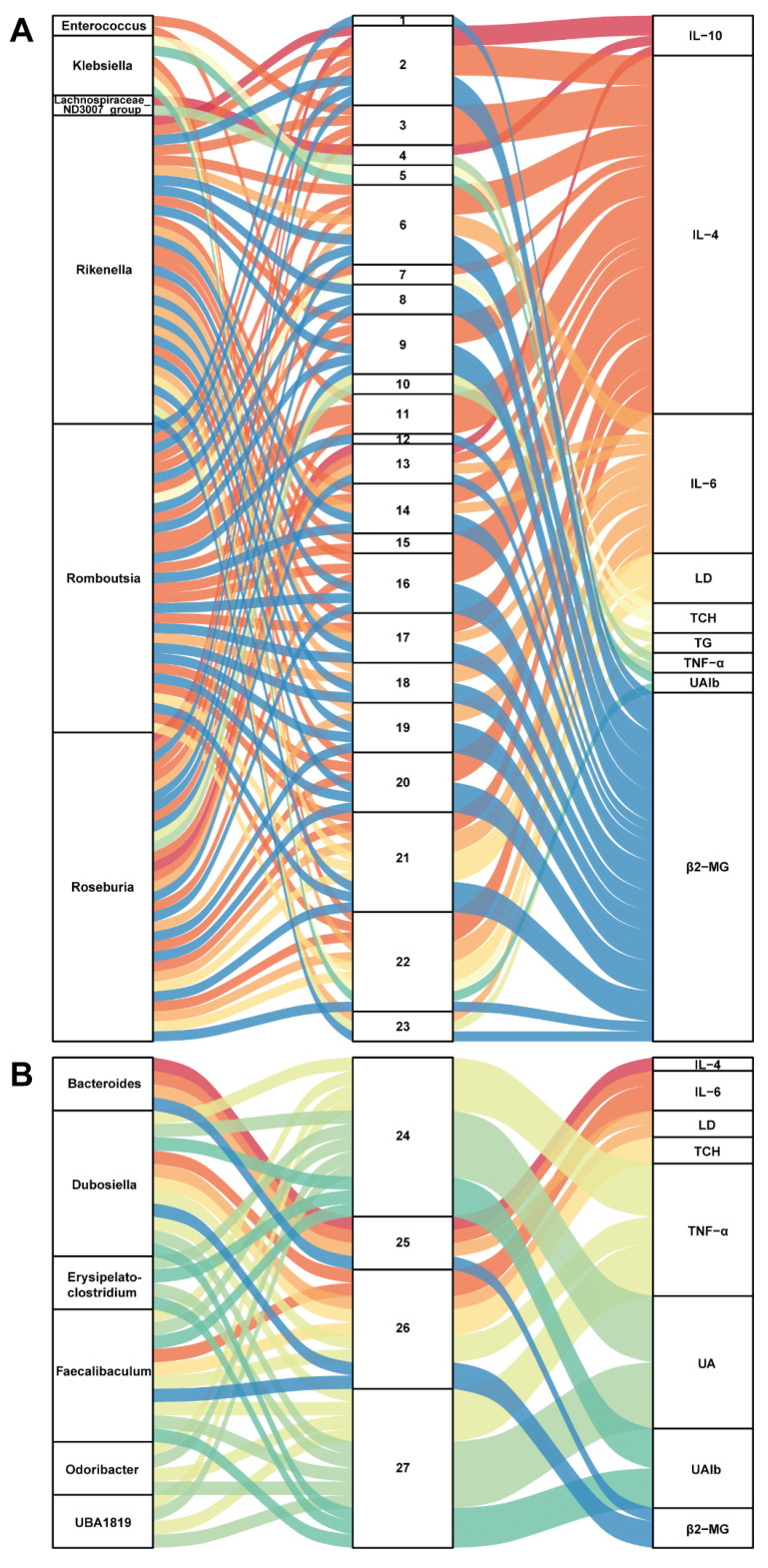
Correlation analysis of gut microbiota, serum metabolites, and biochemical indicators. (**A**,**B**) The Sankey diagrams of the combined analysis of 16sRNA sequencing and untargeted metabolomics. Each number corresponds to a metabolite.

To further build linkage among gut microbiota, serum metabolites, and biochemical indices, we employed correlation analysis of abundances of gut bacterial genera, serum metabolites, and related indices. As presented in Figure 5A, six genera (*Enterococcus*, *Klebsiella*, *Lachnospiraceae_ND3007_group*, *Rikenella*, *Roseburia*, and *Romboutsia*) were positively related to 23 metabolites, which positively correlated with IL-6, LD, TCH, TG, TNF-α, UAlb, and β2-MG but were negatively associated with IL-4 and IL-10. On the contrary, Figure 5B shows that six genera (*Bacteroides*, *Dubosiella*, *Erysipelatoclostridium*, *Odoribacter*, *Faecalibaculum*, and *UBA1819*) were positively associated with 4 metabolites (2-methyl hippuric acid, 3-hydroxy-cis-5-tetradecenoylcarnitine, pentadecanoic acid, and 12-MTA) and then positively related to IL-4 but negatively correlated with IL-6, LD, TCH, TNF-α, UA, UAlb, and β2-MG. These correlations may participate in the protective effect of SZ-A on kidneys in STZ/HFD-conditioned mice. Noteworthily, it is a potential mechanism for SZ-A to protect against DN by regulating the level of pentadecanoic acid.

1,4-(1-adamantyl)-2-methyl-1,3-thiazole; 2, 4-pregnen-17alpha,20alpha-diol-3-one; 3, 5-(4-benzylpiperazino)-2,4-(1H,3H)-pyrimidinedione; 4, 7-(1-pyrrolidinyl)pyrimido[4,5-d]pyrimidin-4-amine; 5, 8-hydroxyquinoline; 6, ACar 20:1; 7, androsterone; 8, cortolone; 9, Dl-2-aminooctanoic acid; 10, DL-dipalmitoylphosphatidylcholine; 11, DNH; 12, estriol; 13, hexanoylcarnitine; 14, hydroxypropionylcarnitine; 15, LysoPE(0:0/18:4(6Z,9Z,12Z,15Z)); 16, N-caffeoyl-putrescine; 17, N6,N6,N6-trimethyl-L-lysine; 18, O-phosphoethanolamine; 19, oxoadipic acid; 20, pregabalin; 21, sphingosine 1-phosphate; 22, succinylcholine; 23, xanthine; 24, 12-methyltetradecanoic acid; 25, 2-methyl hippuric acid; 26, 3-hydroxy-cis-5-tetradecenoylcarnitine; 27, pentadecanoic acid.

### 3.6. Gut Microbiota from SZ-A-Treated DN Mice Reverses the Level of Glucose and Lipid in DN Mice

**Figure 6:**
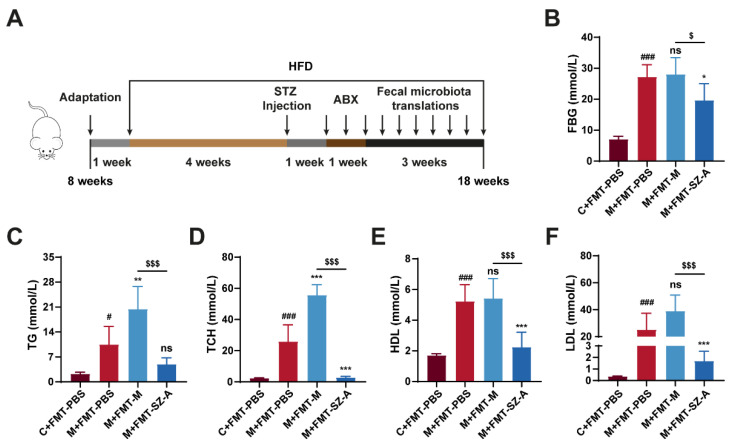
The fecal microbiota transplantation experiment mitigates metabolic disorders in HFD and STZ-induced mice. (**A**) Procedure for antibiotic pretreatment and fecal microbiota transplantation. (**B**) Blood glucose levels. (**C**) Serum TG levels. (**D**) Serum TCH levels. (**E**) Serum HDL levels. (**F**) Serum LDL levels. The data represent the mean ± SD. n = 6, # *p* < 0.05, ### *p* < 0.001 versus the C + FMT-PBS group; * *p* < 0.05, ** *p* < 0.01, *** *p* < 0.001 versus the M + FMT-PBS group; $ *p* < 0.05, $$$ *p* < 0.001 versus the M + FMT-M group; ns, not significant.

To investigate whether the effects of SZ-A on mitigating DN depended on the intestinal microbiota, an FMT experiment was performed. We established the DN model using HFD combined with STZ injection, and then we pretreated mice with antibiotics to deplete the gut microbiota before receiving PBS and intestinal microbiota supernatant from model mice or SZA-treated model mice (Figure 6A). After sacrifice, we detected some biochemical indexes related to glucose and lipid metabolism. PBS-treated model (M + FMT-PBS) mice showed elevated FBG, TCH, TG, HDL, and LDL levels compared with control (C + FMT-PBS) mice, suggesting obvious glucose and lipid metabolism disorders in HFD and STZ-induced mice. Surprisingly, the DN mice that received FMT from SZA-treated DN (M + FMT-SZ-A) mice showed notable reductions in FBG, TCH, TG, HDL, and LDL compared with the M + FMT-PBS group, while these indices were not reversed in the DN mice that received FMT from DN mice (M + FMT-M group) (Figure 6B–F). These data suggest that SZ-A markedly decreases blood glucose and lipid levels and improves metabolism disorders in HFD and STZ-induced mice through the gut microbiota.

### 3.7. Gut Microbiota Mediates the Renal Protective Effect of SZ-A

**Figure 7:**
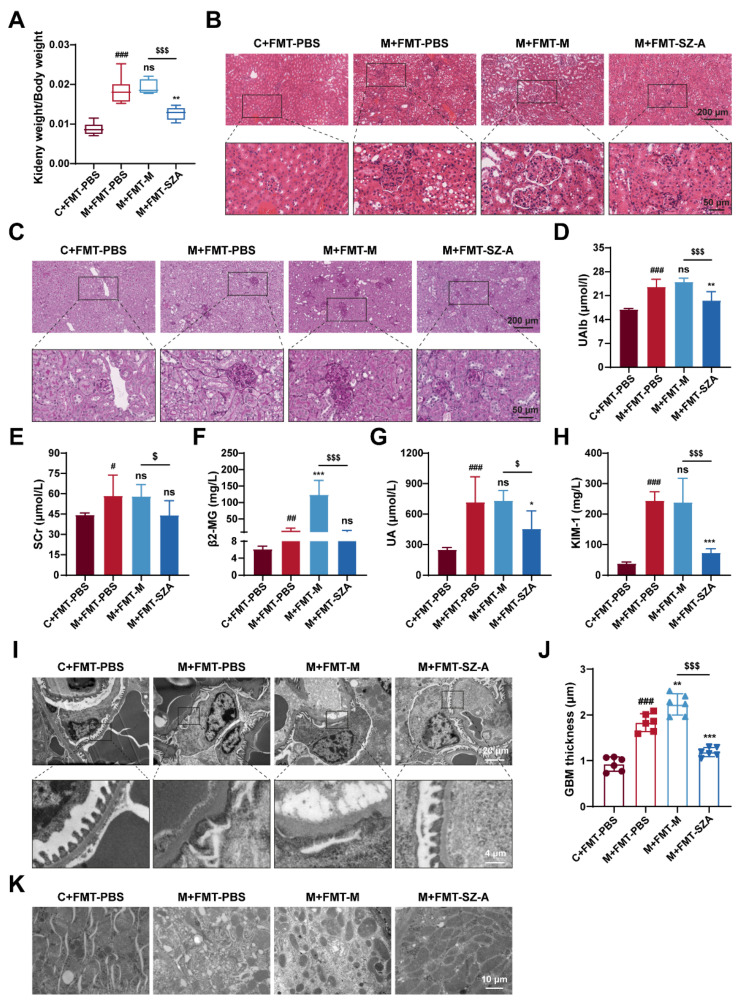
The renoprotection effects of SZ-A were transferable by gut microbiota in HFD and STZ-induced mice. (**A**) Ratio of kidney weight to body weight, n = 6. (**B**) Representative images of hematoxylin–eosin (H&E) staining of kidney tissue sections from each group (scale bars: 200 μm and 50 μm). (**C**) Representative images of PAS staining of kidney tissue sections from each group (scale bars: 200 μm and 50 μm). (**D**) UAlb content, n = 6. (**E**–**H**) Serum biochemical indexes of mice, n = 5–6. (**I**,**J**) Representative electron microscopy images of mouse glomerulus from each group (scale bars: 20 μm and 4 μm) and TEM quantification of GBM thickness, n = 6. (**K**) Representative electron microscopy images of kidney tissue mitochondria from each group (scale bar: 10 μm). The data represent the mean ± SD. # *p* < 0.05, ## *p* < 0.01, ### *p* < 0.001 versus the C + FMT-PBS group; * *p* < 0.05, ** *p* < 0.01, *** *p* < 0.001 versus the M + FMT-PBS group; $ *p* < 0.05, $$$ *p* < 0.001 versus the M + FMT-M group; ns, not significant.

Of note, kidney injury was drastically attenuated in DN mice after being treated with FMT from SZ-A-treated DN mice. As expected, the ratio of kidney weight to body weight in M + FMT-PBS mice was significantly increased, and there was no significant change in M + FMT-M mice, while this value in M + FMT-SZ-A mice was notably reversed and lower than that in the M + FMT-M group (Figure 7A). Histological analysis further revealed obvious protection against mesangial matrix expansion, glomerular hypertrophy, inflammatory infiltration, and glycogen deposition in M + FMT-SZ-A mice (Figure 7B,C). In addition, PAS staining showed that the M + FMT-PBS mice exhibited obvious glycogen deposition, which was predominantly localized to the glomerulus; however, glycogen deposition was clearly ameliorated in the M + FMT-SZA group (Figure 7C). Biochemical parameter analysis also showed the same results. The levels of UAlb, SCr, β2-MG, UA, and KIM-1 significantly increased in M + FMT-PBS mice compared with the C + FMT-PBS group; however, these indices decreased in M + FMT-SZ-A mice in contrast with the M + FMT-PBS group or the M + FMT-M group (Figure 7D–H).

Next, we further observed the ultrastructure of the kidney tissue by TEM. Foot process fusion occurred and the thickness of GBM markedly increased, which was aggravated in the M-FMT-M group; however, these ultrastructure changes were alleviated in the M + FMT-SZ-A group (Figure 7I,J). Similarly, mitochondria dissolved and decreased in the M + FMT-PBS group and M + FMT-M group; however, the morphology and number of mitochondria in the M + FMT-SZ-A group were effectively attenuated (Figure 7K). Given that, the gut microbiota of SZ-A-treated DN mice demonstrates substantial protection against renal injury.

### 3.8. Pentadecanoic Acid Produced by Gut Microbiota Ameliorates Podocyte Apoptosis In Vitro

**Figure 8:**
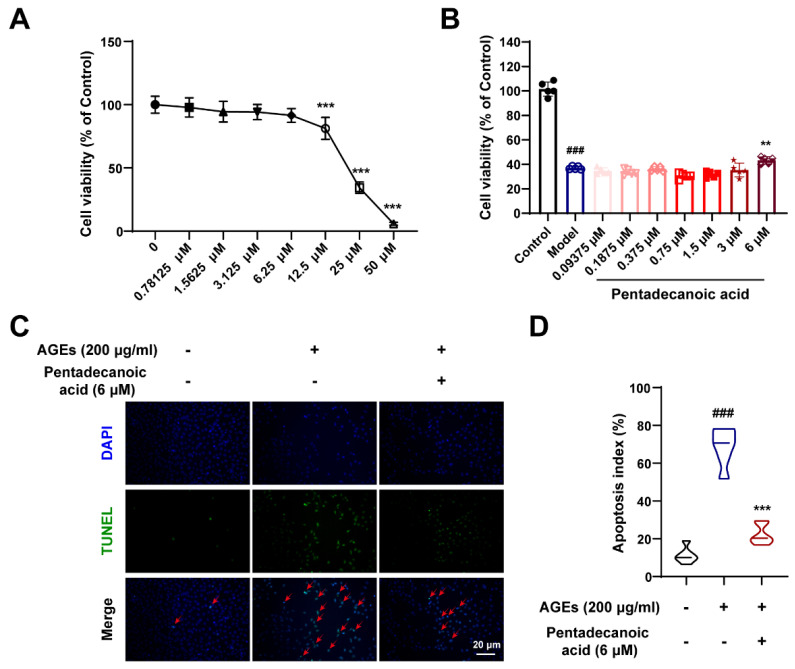
Pentadecanoic acid restored podocyte injury induced by AGEs. (**A**) Cytotoxicity of pentadecanoic acid, n = 6. (**B**) The survival rate of MPC5 cells induced by AGEs under different concentrations of pentadecanoic acid, n = 5. (**C**,**D**) Representative TUNEL staining images of MPC5 cells (scale bar: 20 μm) and quantification of apoptosis, n = 6. The data represent the mean ± SD. ### *p* < 0.001; ** *p* < 0.01, *** *p* < 0.001.

To further verify the protective effects of gut microbiota metabolites, we performed in vitro experiments. As shown in Figure 8A, we explored the cytotoxicity of pentadecanoic acid. When the concentration of pentadecanoic acid is lower than 6.25 μM, it has no obvious toxic effect on MPC5 cells (Figure 8A). Therefore, we studied the protective effect of pentadecanoic acid at a concentration of 0–6 μM on podocytes. After inducing MPC5 cells with 200 μg/mL AGEs, the cell viability was significantly reduced; however, pentadecanoic acid significantly restored the cell survival rate at 6 μM (Figure 8B). In addition, TUNEL assay results demonstrated that the AGEs-induced apoptosis index was significantly increased; however, pentadecanoic acid could notably improve podocyte apoptosis (Figure 8C,D). In general, the intestinal flora metabolite pentadecanoic acid can protect podocytes from apoptosis caused by AGEs. These data indicate that SZ-A may protect podocytes against apoptosis by increasing the level of pentadecanoic acid.

## 4. Discussion

Currently, despite the strict control of blood glucose and improvement of renal function in patients with DN, it is still the main cause of end-stage renal diseases. In this case, novel avenues are desperately needed to prevent the progression of DN towards end-stage renal failure. To establish the DN animal model for our study, we injected STZ into mice after feeding them a high-fat diet for 4 weeks. In vivo experiments showed that SZ-A ameliorated kidney injury in HFD- and STZ-induced mice. Through multi-omics analysis, we found that SZ-A altered gut microbiota composition in DN progression. In particular, SZ-A-treated DN mice exhibited a considerable increase in the abundance of *Dubosiella* and *Lactobacillus* as well as an upregulation of the metabolite pentadecanoic acid. The gut microbiota of DN mice treated with SZ-A protected diabetic kidney disease animals against renal injury, as confirmed by the FMT experiment. Our work provided evidence that SZ-A counteract DN progression by regulating the gut microbiota.

Long-term hyperglycemia and hyperlipidemia have been linked to the formation and progression of DN. Concretely, hyperglycemia exacerbates renal damage by triggering glomerular hyperfiltration and a host of intercellular responses, such as inflammation and oxidative stress [24]. Meanwhile, lipid accumulation has a lipotoxic effect on the kidney, which not only leads to podocyte dysfunction and apoptosis but also correlates with glomerulosclerosis and renal tubulointerstitial injury [25,26]. Surprisingly, we found that SZ-A treatment obviously reduced blood glucose and lipid levels in the model group, which is consistent with recent research [27,28,29], implying the potential value of SZ-A in DN treatment.

In the progression of DN, there are numerous pathological features, such as inflammatory infiltration, glomerular hypertrophy, GBM thickening, and podocyte injury [30]. Urine contains more microalbumin as a result of these lesions, which impair the filtration and reabsorption functions of the kidney [31]. Furthermore, abnormal levels of urea nitrogen, creatinine, and other factors in serum are important indicators of renal damage [32,33,34]. In line with this literature, our current research discovered that the model mice had significantly higher levels of urinary microalbumin and serum UA, SCr, BUN, and β2-MG. However, supplementation with SZ-A reversed these changes in a dose-dependent manner. As predicted, SZ-A obviously alleviate DN, as evidenced by improved glomerular morphology, decreased albuminuria, mesangial cell proliferation, glycogen deposition, and GBM thickness (Figure 2). These findings are in conformity with one previous study in which SZ-A ameliorate renal function in Zucker diabetic fatty rats in a dose-dependent manner [35].

Emerging evidence indicates a crucial role of the gut-kidney axis in DN development [36]. Preclinical and clinical studies have displayed that diabetes remarkably shifts the composition of the gut microbiota in DN patients and animals compared to controls [19,37]. The imbalance of the intestinal microbiota destroys the intestinal tight junction, leading to the leakage of intestinal flora metabolites and toxic substances into the bloodstream, which causes damage to the kidney [38]. A variety of natural active substances can improve renal injury by regulating the composition and metabolism of the gut microbiota [39]. Given that SZ-A delay the absorption of carbohydrates in the small intestine [40,41], we speculated that SZ-A ameliorate DN by modulating the composition and metabolic profiles of the gut microbiota. As demonstrated by our 16S rRNA sequencing data, the composition of the gut microbiota in DN mice changed significantly following SZ-A treatment. Several bacteria, including *Blautia*, *Enterococcus*, *Lachnospiraceae_NK4A136_group*, *Mucispirillum*, *Desulfovibrio*, *Acetatifactor*, *Helicobacter*, *Roseburia*, *Romboutsia*, and *Rikenella*, became less abundant at the genera level; in contrast, the percentage of *Bacteroides*, *Dubosiella*, *Alistipes*, *Erysipelatoclostridium*, *Odoribacter*, *Faecalibaculum*, *UBA1819*, and *Lactobacillus* at the genus level rose in DN mice after SZ-A administration.

In line with our studies, Liu et al. revealed that SZ-A could reduce the abundance of *Desulfovibrio* while increasing the abundance of *Bacteroides* and *Faecalibaculum* in diabetic KKAy mice [8]. Additionally, it has been reported that *Blautia* is associated with obesity and elevated UAlb by producing SCFAs [42,43]. Similarly, our study found that SZ-A reduced the abundance of *Blautia* in DN mice. Moreover, SZ-A increased the level of *Alistipes*, a member of the *Rickenellaceae* family, which has a negative correlation with obesity by modulating fat absorption in the gut [44]. Furthermore, the increase in *Lactobacillus* and *Dubosiella* was most notable, which is consistent with previous reports. *Lactobacillus* participates in intestinal mucosal barrier repair and protects the intestinal epithelium from malignant bacteria invasion, which is negatively linked with factors related to hyperglycemia and kidney injury [45,46,47,48]. *Dubosiella* plays an anti-inflammatory role in colitis and is closely associated with ferroptosis [49]. Our findings elucidated that *Dubosiella* had a positive correlation with anti-inflammatory factors and a negative link with pro-inflammatory factors. Given the above, SZ-A treatment significantly raised the abundance of beneficial bacteria and reduced the level of unfavorable bacteria. To further confirm the role of gut microbiota, we depleted the microbiota with antibiotics in DN mice and transferred fecal bacteria supernatant from DN mice either with or without SZ-A treatment. Recolonization of intestinal bacteria from SZ-A-treated DN mice led to intriguing reversals of renal function, glomerular morphology, and ultrastructure changes in the kidney, unveiling that SZ-A possess renoprotective benefits in a gut microbiota-dependent manner.

In recent years, growing evidence has elucidated that taking more dietary fiber relieves obesity, diabetes, and diabetic kidney disease [50,51,52]. Commensal bacteria promote the fermentation of dietary fibers and the production of SCFAs to maintain the gastrointestinal barrier and immune homeostasis [53]. Other than SCFAs, both medium-chain fatty acids and long-chain fatty acids, although with few studies, also play pivotal roles in regulating energy metabolism [54]. Interestingly, we found that *Dubosiella* is associated with four metabolites, including 2-methyl hippuric acid, 3-hydroxy-cis-5-tetradecenoylcarnitine, pentadecanoic acid, and 12-MTA. Among these, pentadecanoic acid is a type of dairy-specific saturated fatty acid, and numerous studies have reported its beneficial effects on disease progression [54]. A recent study has demonstrated that *P. distasonis* directly synthesizes pentadecanoic acid in vivo and in vitro. By inhibiting the expression of inflammatory factors and TG synthesis regulators in the liver of non-alcoholic steatohepatitis mice, this metabolite maintains intestinal barrier integrity and inhibits intestinal inflammation [55]. Cheng et al. proved its anti-fibrotic role in attenuating lung fibrosis [56]. As expected, we validated that pentadecanoic acid inhibits podocyte apoptosis and plays renal protective roles in vitro. Based on the above data, we concluded that pentadecanoic acid could function as a renoprotective metabolite in DN and that SZ-A might increase levels of it by regulating the gut microbiota composition. Additionally, DN is asymptomatic in the early stages and has been in the late stage when urinary protein can be detected in DN patients in clinical settings [57]. Our work offers a novel strategy for the diagnosis and treatment of DN since intestinal microbiota metabolites have a strong relationship with DN.

However, our research presents some limitations that need to be addressed in further study. We screened a large number of altered metabolites of the gut microbiota under SZ-A supplementation conditions; nonetheless, in vivo experiments to explore the effects of these metabolites as well as related targets remain necessary and attractive. Furthermore, the absence of further mechanism studies is our shortcoming. The issues to be elucidated above will be the focus of our future work.

## 5. Conclusions

In summary, our study finds that SZ-A attenuate lipid and glucose metabolic disorders as well as kidney injury in the DN mice. Notably, the potential mechanism is revealed from multi-omics combination analysis and FMT experiments, by which SZ-A ameliorate gut microbiota disorders through elevating the abundance of beneficial bacteria *Dubosiella* and the level of beneficial metabolite pentadecanoic acid. Our findings confirm the hypothesis that SZ-A protect against DN through modulating the gut microbiota, highlighting that SZ-A are a promising drug for DN treatment that deserves further research.

## Data Availability

The data of this study are available from the corresponding author upon reasonable request. The data are not publicly available due to privacy reasons.

## Abbreviations

DN, diabetic nephropathy; SZ-A, *Ramulus Mori* alkaloids; FMT, fecal microbiota transplantation; HFD, high-fat diet; SCFAs, short-chain fatty acids; FBG, fasting blood glucose; TCH, total cholesterol; TG, triglycerides; BUN, blood urea nitrogen; SCr, serum creatinine; IL-6, interleukin-6; TNF-α, tumor necrosis factor alpha; STZ, streptozotocin; KIM-1, kidney injury molecule-1; UAlb, urinary microalbumin; UA, uric acid; β2-MG, beta-2-microglobulin; H&E, hematoxylin and eosin; PCoA, principal coordinate analysis; IL-10, interleukin 10; PLS-DA, partial least-squares discriminant analysis; 12-MTA, 12-methyltetradecanoic acid; TEM, transmission electron microscope; GBM, glomerular basement membrane; HDL, high-density lipoprotein; LDL, low-density lipoprotein; HPLC-MS/MS, high-performance liquid chromatography-tandem mass spectrometry.
